# Construction of a novel circRNA-miRNA-ferroptosis related mRNA network in ischemic stroke

**DOI:** 10.1038/s41598-023-41028-1

**Published:** 2023-09-12

**Authors:** Huirong Xie, Yijie Huang, Yanli Zhan

**Affiliations:** 1 Department of Neurology, Lishui Municipal Central Hospital, Lishui Hospital of Zhejiang University, The Fifth Affiliated Hospital of Wenzhou Medical University, Lishui Clinical Research Center for Neurological Diseases, 289 Kuocang Road, Lishui, 323000 Zhejiang China; 2 Cerebrovascular Research Laboratory, Lishui Municipal Central Hospital, Lishui Hospital of Zhejiang University, The Fifth Affiliated Hospital of Wenzhou Medical University, Lishui Clinical Research Center for Neurological Diseases, 289 Kuocang Road, Lishui, 323000 Zhejiang China

**Keywords:** Molecular biology, Neuroscience

## Abstract

Molecule alterations are important to explore the pathological mechanism of ischemic stroke (IS). Ferroptosis, a newly recognized type of regulated cell death, is related to IS. Identification of the interactions between circular RNA (circRNA), microRNA (miRNA) and ferroptosis related mRNA may be useful to understand the molecular mechanism of IS. The circRNA, miRNA and mRNA transcriptome data in IS, downloaded from the Gene Expression Omnibus (GEO) database, was used for differential expression analysis. Ferroptosis related mRNAs were identified from the FerrDb database, followed by construction of circRNA-miRNA-ferroptosis related mRNA network. Enrichment and protein–protein interaction analysis of mRNAs in circRNA-miRNA-mRNA network was performed, followed by expression validation by reverse transcriptase polymerase chain reaction and online dataset. A total of 694, 41 and 104 differentially expressed circRNAs, miRNAs and mRNAs were respectively identified in IS. Among which, dual specificity phosphatase 1 (DUSP1), nuclear receptor coactivator 4 (NCOA4) and solute carrier family 2 member 3 (SLC2A3) were the only three up-regulated ferroptosis related mRNAs. Moreover, DUSP1, NCOA4 and SLC2A3 were significantly up-regulated in IS after 3, 5 and 24 h of the attack. Based on these three ferroptosis related mRNAs, 4 circRNA-miRNA-ferroptosis related mRNA regulatory relationship pairs were identified in IS, including hsa_circ_0071036/hsa_circ_0039365/hsa_circ_0079347/hsa_circ_0008857-hsa-miR-122-5p-DUSP1, hsa_circ_0067717/hsa_circ_0003956/hsa_circ_0013729-hsa-miR-4446-3p-SLC2A3, hsa_circ_0059347/hsa_circ_0001414/hsa_circ_0049637-hsa-miR-885-3p-SLC2A3, and hsa_circ_0005633/hsa_circ_0004479-hsa-miR-4435-NCOA4. In addition, DUSP1 is involved in the signaling pathway of fluid shear stress and atherosclerosis. Relationship of regulatory action between circRNAs, miRNAs and ferroptosis related mRNAs may be associated with the development of IS.

## Introduction

Stroke, a common vascular disease, causes disability and death^1^. Approximately 87% of all stroke cases are ischemic stroke (IS)^2^. IS leads to ischemic hypoxic necrosis of local tissues and corresponding neurological defects^3^. The main clinical symptoms of IS include sudden fainting, lateral appendage numbness, hemiplegia and aphasia, which may be life-threatening^4,5^. Therefore, elucidating potential pathological mechanism of IS, especially molecular mechanism, is urgent.

Some biological molecules may be involved in IS, such as circular RNAs (circRNAs), microRNAs (miRNAs) and mRNAs. CircRNAs, endogenous single-stranded RNA molecules, forms a circle through a covalent binding^1^. CircRNAs display a range of regulatory functions in RNA biology. Some circRNAs function as sponges that sequester miRNAs, regulating the expression of target mRNAs^6^. MiRNA modulates protein synthesis by binding to the 30 untranslated regions of protein-coding mRNA transcripts, which result in translational repression^7^. The circRNA-miRNA-mRNA axis plays roles in numerous biological processes^8,9^. It is found that hsa_circ_0000607 plays an important role in acute IS by regulating the hsa-miR-337-3p/BCL2 apoptosis regulator (Bcl2) axis^10^.

Beside biological molecules, some biological process may be involved in IS. During ischemia, the disruption of blood supply to brain tissues will promote a cascade of patho-physiological responses, such as ferroptosis^11^. It has demonstrated that ferroptosis is involved in many diseases, including IS^12,13^. It is found that ferroptosis plays a key role in IS through influencing iron metabolism, whereas inhibiting ferroptosis reverses ischemic damages^13–15^. Understanding the role of ferroptosis in IS may throw some light on the development of methods for diagnosis and treatment of this devastating disease. In this study, we aimed to identify differentially expressed circRNAs, miRNAs, ferroptosis related mRNAs, followed by the construction of circRNAs-miRNAs-ferroptosis related mRNAs network in IS.

## Material and methods

### Dataset search

All data were retrieved from the Gene Expression Omnibus (GEO) database. Search keywords were (“stroke” [All Fields]) AND “Homo sapiens”[porgn] AND “gse”[Filter]. The datasets that met the following criteria were included: (1) these datasets must be genome-wide circRNA/miRNA/mRNA transcriptome data; (2) these data were collected from the blood of the IS case group and the normal control group; (3) standardized or original datasets were considered. A total of 1 circRNA dataset (GSE133768), 1 miRNA dataset (GSE199942) and 1 mRNA dataset (GSE16561) were included in this study. Detailed information of the above three datasets is shown in Table 1.

**Table 1:** Detailed information of 3 datasets of IS.

GEO accession	Author	Platform	Samples (NC: IS)	Year	Sample
GSE133768	Lei Zuo	GPL21825 074,301 Arraystar Human CircRNA microarray V2	3: 3	2020	Blood
GSE199942	Junli Hao	GPL16791Illumina HiSeq 2500 (Homo sapiens)	5: 5	2022	Blood
GSE16561	Taura L Barr	GPL6883Illumina HumanRef-8 v3.0 expression beadchip	24: 39	2010	Blood

*NC* normal controls, *IS* ischemic stroke.

### Identification of differentially expressed circRNAs, miRNAs and mRNAs in IS

In datasets, the probes corresponding to multiple circRNAs/miRNAs/mRNAs were removed. Those circRNAs/miRNAs/mRNAs corresponding to multiple probes were averaged as the expression level. After scale standardization and logarithmic processing of datasets, the “Limma” software package in R software was used to identify differentially expressed circRNAs, miRNAs and mRNAs under the screening criteria of |log_2_ fold change (FC)|> 1 and *p* value < 0.05. Volcano map and heat map were generated using the “ggplot2” and “pheatmap” packages in R software.

### Construction of circRNA-miRNA-ferroptosis related mRNA network in IS

The circbank was utilized to predict the negatively regulated miRNAs of differentially expressed circRNAs. The intersection of the predicted miRNAs and differentially expressed miRNAs was obtained. The miRWalk was used to identify negatively regulated mRNAs of differentially expressed miRNAs. The intersection of predicting mRNAs and differentially expressed mRNAs was identified. The circRNA-miRNA targeting relationship pair and miRNA-mRNA targeting relationship pair were integrated to construct circRNA-miRNA-mRNA (ceRNA) network. In addition, ferroptosis related mRNAs were identified from the FerrDb database. The intersection of the ferroptosis related mRNAs and differentially expressed mRNAs in ceRNA network was obtained, followed by construction of circRNA-miRNA-ferroptosis related mRNA network.

### Function and protein–protein interaction (PPI) analysis of mRNAs in the ceRNA network in IS

Firstly, the David database was used for Gene Ontology (GO) and Kyoto Encyclopedia of Genes and Genomes (KEGG) of mRNAs in the ceRNA network. KEGG contains numerous signaling pathways^16–18^. Significantly enriched GO and KEGG terms were screened out under the screening criteria of *p* value < 0.05. Secondly, in order to investigate the interaction of proteins encoded by mRNAs in the ceRNA network, PPI analysis was performed via string database.

### Expression validations of circRNAs, miRNAs and mRNAs in IS by reverse transcriptase polymerase chain reaction (RT-PCR) and online dataset

Firstly, RT-PCR was utilized to validate the expression of circRNAs, miRNAs and ferroptosis related mRNAs in blood samples form IS patients. The inclusion criteria of IS patients were as follows: (1) first-episode patients were diagnosed based on head computed tomography (CT) (or magnetic resonance imaging (MRI)) and clinical diagnostic criteria; (2) the age range of the patients was 18 to 75 years; (3) patients had no obvious neurological damage caused by cerebrovascular or other causes before onset; (4) patients had no previous history of stroke and no lesion. The exclusion criteria of IS patients were as follows: (1) patients with cardiogenic cerebral embolism, transient ischemic attack, hemorrhagic infarction, occult cerebrovascular malformation, and traumatic cerebrovascular disease; (2) patients had neurological deficits caused by non-neurovascular causes such as malignant tumors, severe pulmonary infections, severe heart, liver and renal insufficiency, and hemorrhagic diseases; (3) patients had serious mental illness; (4) patients had incomplete clinical data. The inclusion criteria of healthy individuals were as follows: (1) these individuals matched the frequency of the IS patients by gender and age; (2) these individuals were over 18 years old; (3) these individuals had no history of stroke or other cardiovascular or cerebrovascular disease. The exclusion criteria of healthy individuals were as follows: (1) those individuals with previous history of stroke, head trauma and surgery, or neurological diseases; (2) those individuals were experiencing pregnancy or breastfeeding. Based on the above inclusion and exclusion criteria, a total of 10 IS patients and 9 healthy individuals were enrolled in the present study. Clinical information of 10 IS patients and 9 healthy individuals are listed in Table 2. The blood samples of these individuals were collected for RT-PCR. Glyceraldehyde-3-phosphate dehydrogenase (GAPDH), actin beta (ACTB) and hsa-U6 were used as internal references. All methods were carried out in accordance with relevant local/national/international institutional guidelines and regulations. The study was approved by the ethics committee of the local hospital. Additionally, all individuals provided the informed consent of the patients and their families. In addition, to validate the expression of ferroptosis related mRNAs in IS, GSE58294 dataset (involved blood samples) was selected for analysis. The dataset includes 69 IS cases (at 3, 5 and 24 h after the IS) and 23 normal controls.

**Table 2:** Clinical information of 10 IS patients and 9 healthy individuals in RT-PCR.

Group	Number	Sex	Age (years)	Height (cm)	Weight (kg)	Hypertension	Diabetes	Hyperlipemia	Coronary heart disease	Malignant tumor	Intracranial infection	Smoking history	Drinking history	Family genetic history	History of carotid artery stenting
IS	1	Male	62	158	55	Yes	0	Yes	No	No	No	Yes	No	No	No
	2	Male	43	171	80	Yes	Yes	Yes	No	No	No	Yes	Yes	No	No
	3	Male	68	165	60	Yes	Yes	No	No	No	No	Yes	Yes	No	No
	4	Male	67	168	68.5	No	No	No	No	No	No	Yes	No	No	No
	5	Female	70	155	55	No	Yes	Yes	No	No	No	No	No	No	No
	6	Female	62	155	52	Yes	Yes	No	No	No	No	No	No	No	No
	7	Male	67	164	82	Yes	Yes	No	No	No	No	Yes	Yes	No	No
	8	Female	52	158	60	No	No	No	No	No	No	No	No	No	No
	9	Male	57	168	75	Yes	Yes	Yes	No	No	No	No	Yes	No	No
	10	Female	75	155	53	Yes	Yes	Yes	No	No	No	No	No	No	No
NC	1	Female	64	155	60	No	No	No	No	No	No	No	No	No	No
	2	Female	65	165	62	No	No	No	No	No	No	No	No	No	No
	3	Male	65	167	67	No	No	No	No	No	No	No	Yes	No	No
	4	Female	58	152	50.5	No	No	No	No	No	No	No	Yes	No	No
	5	Male	61	170	70	No	No	No	No	No	No	No	No	No	No
	6	Male	60	162	65	No	No	No	No	No	No	No	No	No	No
	7	Male	54	165	80	No	No	No	No	No	No	Yes	Yes	No	No
	8	Female	64	159	63	No	No	No	No	No	No	No	No	No	No
	9	Female	54	155	61	No	No	No	No	No	No	No	No	No	No

*IS* ischemic stroke, *NC* normal controls.

### Statistical analysis

R software (version 4.0.5) was used for statistical analysis. The Student t test was used to analyze the RT-PCR results and to verify differences in molecule expression. Analysis of variance was used to analyze differences in the online dataset. *P* < 0.05 was considered as statistical difference.

### Ethics approval

The study was approved by the ethics committee of Lishui Municipal Central Hospital Hospital.

### Consent to participate

All individuals provided the informed consent of the patients and their families.

## Results

### Differentially expressed circRNAs, miRNAs and mRNAs in IS

**Figure 1:**
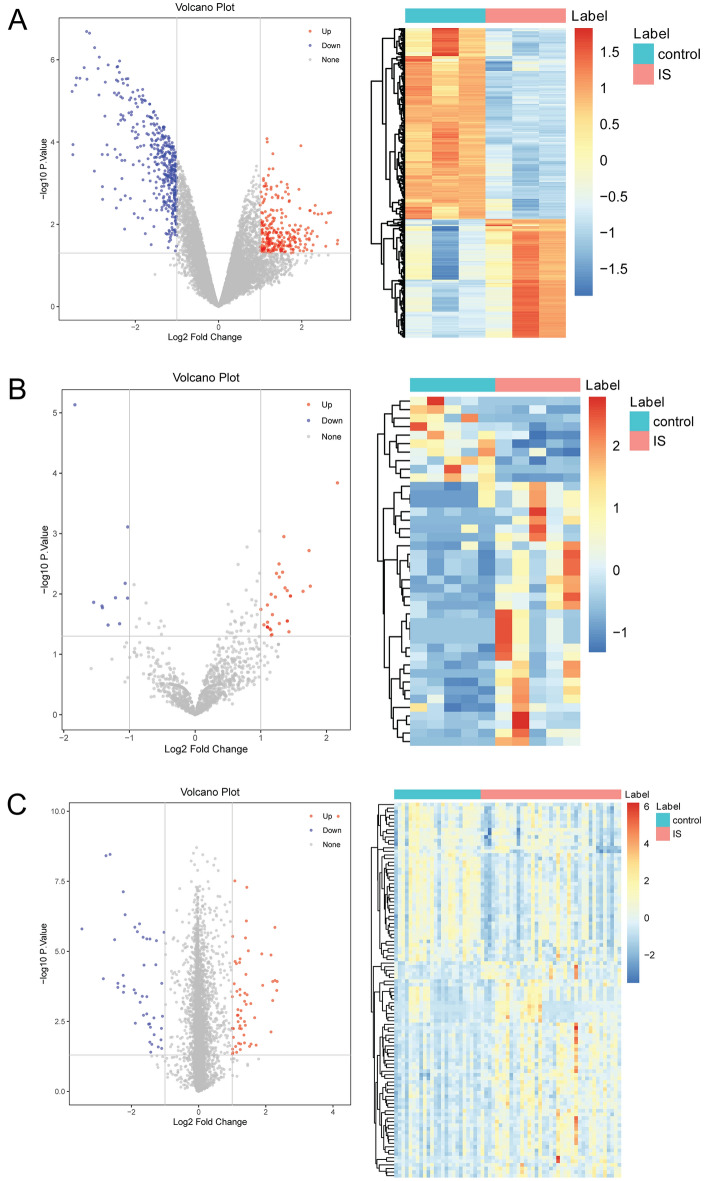
The Volcano map and heat map of all circRNAs (**A**), miRNAs (**B**) and mRNAs (**C**) in IS. IS: ischemic stroke.

A total of 694 (266 up-regulated and 428 down-regulated) differentially expressed circRNAs, 41 (31 up-regulated and 10 down-regulated) differentially expressed miRNAs and 104 (60 up-regulated and 44 down-regulated) differentially expressed mRNAs were identified in IS. The Volcano map and heat map of all circRNAs, miRNAs and mRNAs are presented in Fig. 1A–C, respectively.

### Construction of circRNA-miRNA-ferroptosis related mRNA network in IS

**Figure 2:**
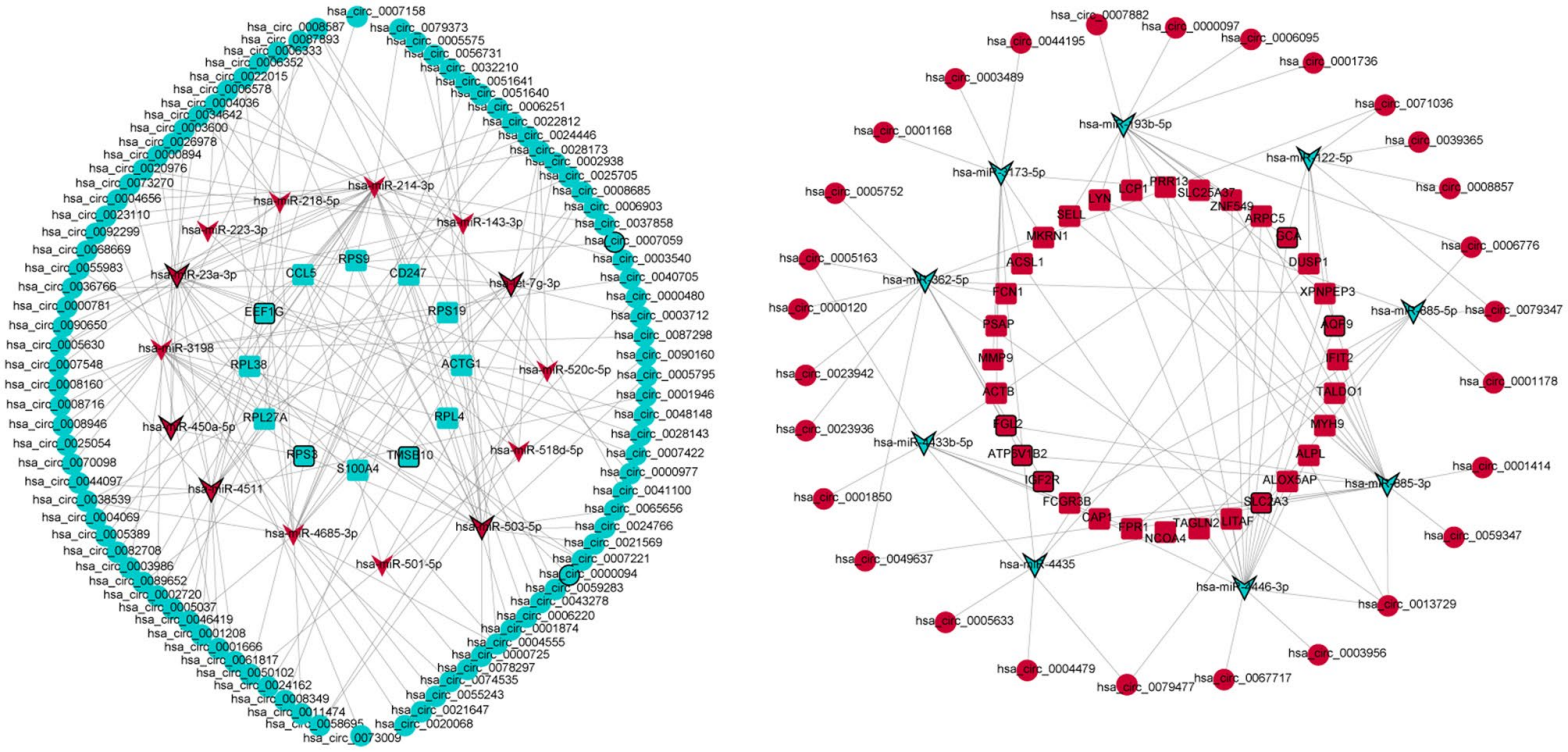
The 121 circRNAs-23 miRNAs-44 mRNAs network in IS. Circle, V shape, and rectangle represent circRNA, miRNA and mRNA, respectively. Red and green color indicates up-regulation and down-regulation, respectively. Black border represents top10 up-regulated and down-regulated circRNA, miRNA and mRNA, respectively.

**Figure 3:**
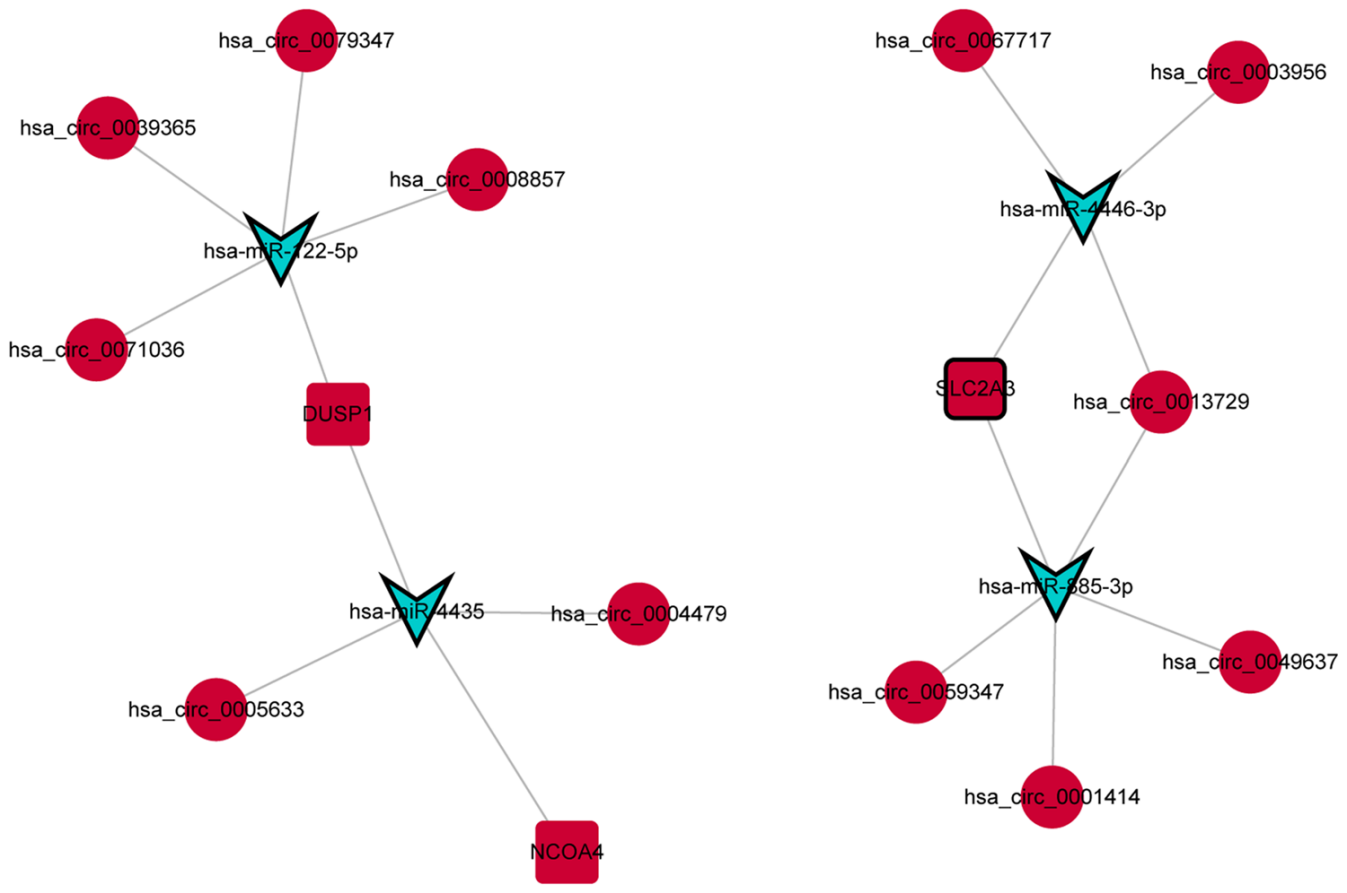
The 12 circRNAs-4 miRNAs-3 ferroptosis related mRNAs network in IS. Circle, V shape, and rectangle represent circRNA, miRNA and mRNA, respectively. Red and green color indicates up-regulation and down-regulation, respectively. Black border represents top10 up-regulated and down-regulated circRNA, miRNA and mRNA, respectively.

Totally, 299 negatively regulated circRNA-miRNA targeting pairs were identified in IS, involving 161 circRNAs and 41 miRNAs. Totally, 103 negatively regulated miRNA-mRNA targeting pairs were identified in IS, involving 24 miRNA and 45 mRNAs. After the integration of 299 negatively regulated circRNA-miRNA targeting pairs and 103 negatively regulated miRNA-mRNA targeting pairs, a ceRNA network was constructed, involving 121 circRNAs, 23 miRNAs and 44 mRNAs (Fig. 2). There are 259 ferroptosis related mRNAs in the FerrDb database. After taking the intersection between 259 ferroptosis related mRNAs and 44 differentially expressed mRNAs in the ceRNA network, 3 common up-regulated mRNAs was identified, including dual specificity phosphatase 1 (DUSP1), nuclear receptor coactivator 4 (NCOA4) and solute carrier family 2 member 3 (SLC2A3). The network of circRNA-miRNA-3 ferroptosis related mRNA network was constructed (Fig. 3), involving 12 up-regulated circRNAs and 4 down-regulated miRNAs. Some circRNA-miRNA-ferroptosis related mRNA regulatory relationship pairs were identified, such as hsa_circ_0071036/hsa_circ_0039365/hsa_circ_0079347/hsa_circ_0008857-hsa-miR-122-5p-DUSP1, hsa_circ_0005633/hsa_circ_0004479-hsa-miR-4435-NCOA4, hsa_circ_0067717/hsa_circ_0003956/hsa_circ_0013729-hsa-miR-4446-3p-SLC2A3 and hsa_circ_0059347/hsa_circ_0001414/hsa_circ_0049637-hsa-miR-885-3p-SLC2A3.

### Function and PPI analysis of mRNAs in the ceRNA network in IS

**Figure 4:**
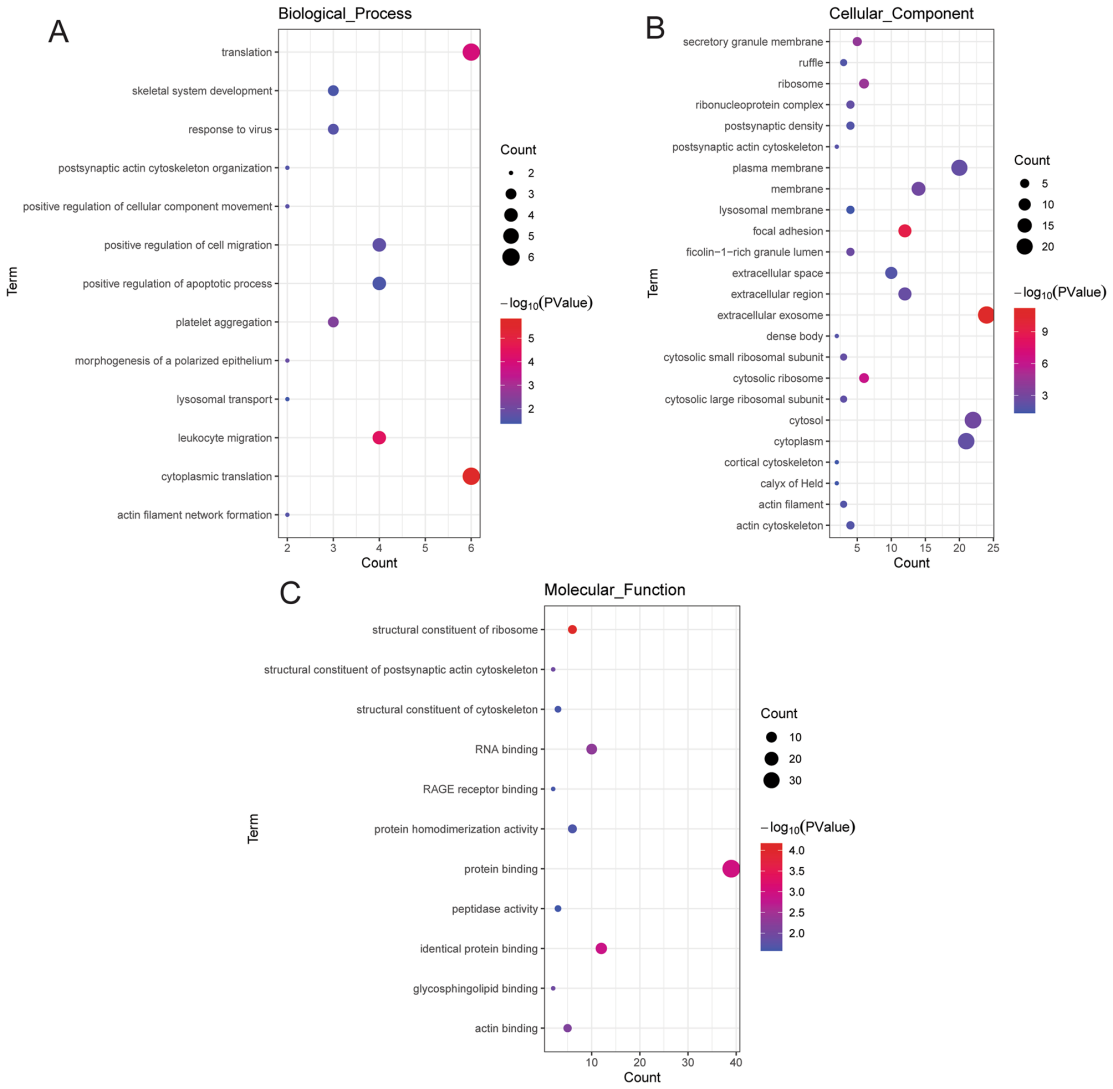
GO functional analysis of mRNAs in the circRNA-miRNA-mRNA network in IS. (**A**) biological process; (**B**) cytological component; C: molecular function.

**Figure 5:**
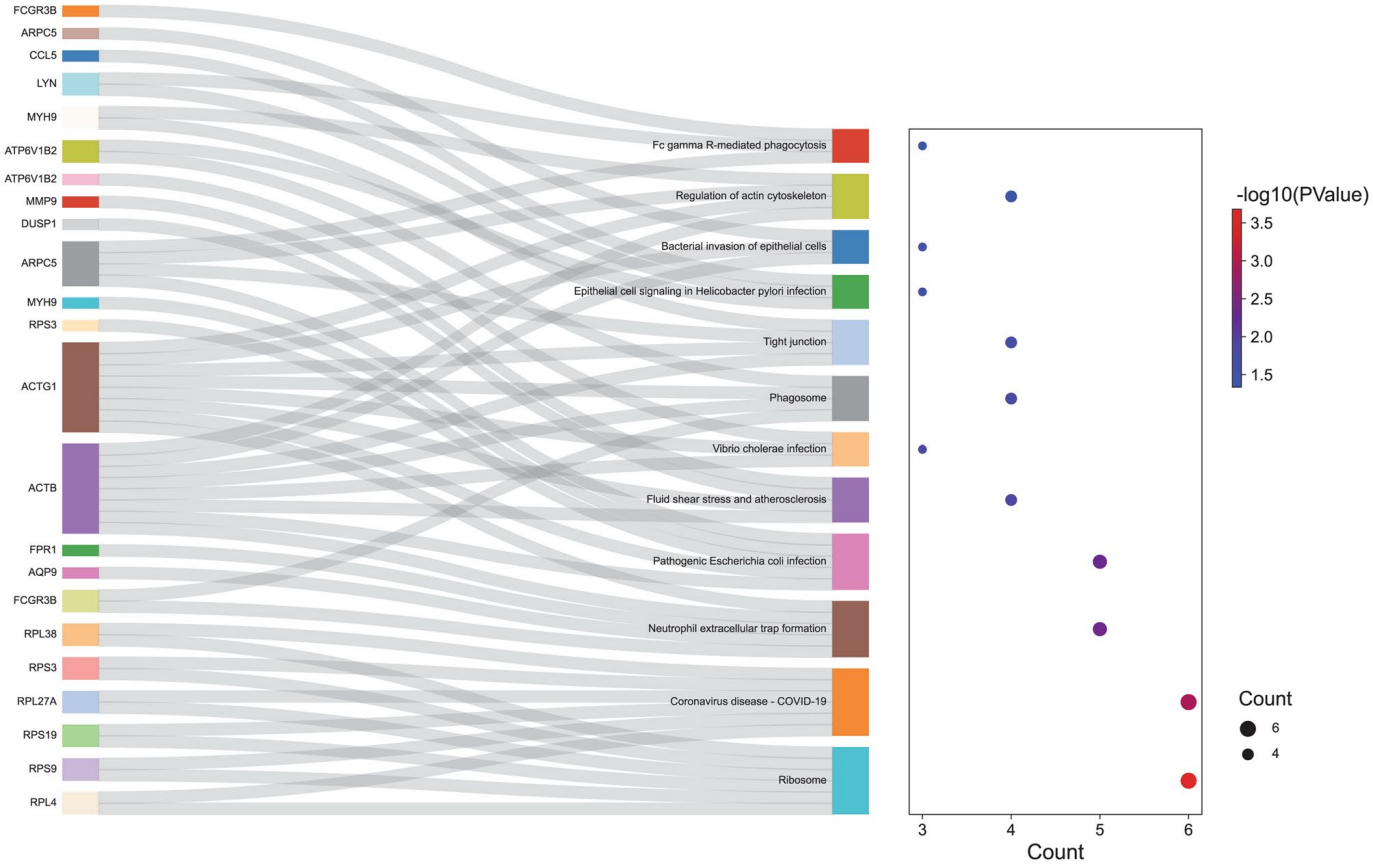
KEGG functional analysis^16–18^ of mRNAs in the circRNA-miRNA-mRNA network in IS.

**Figure 6:**
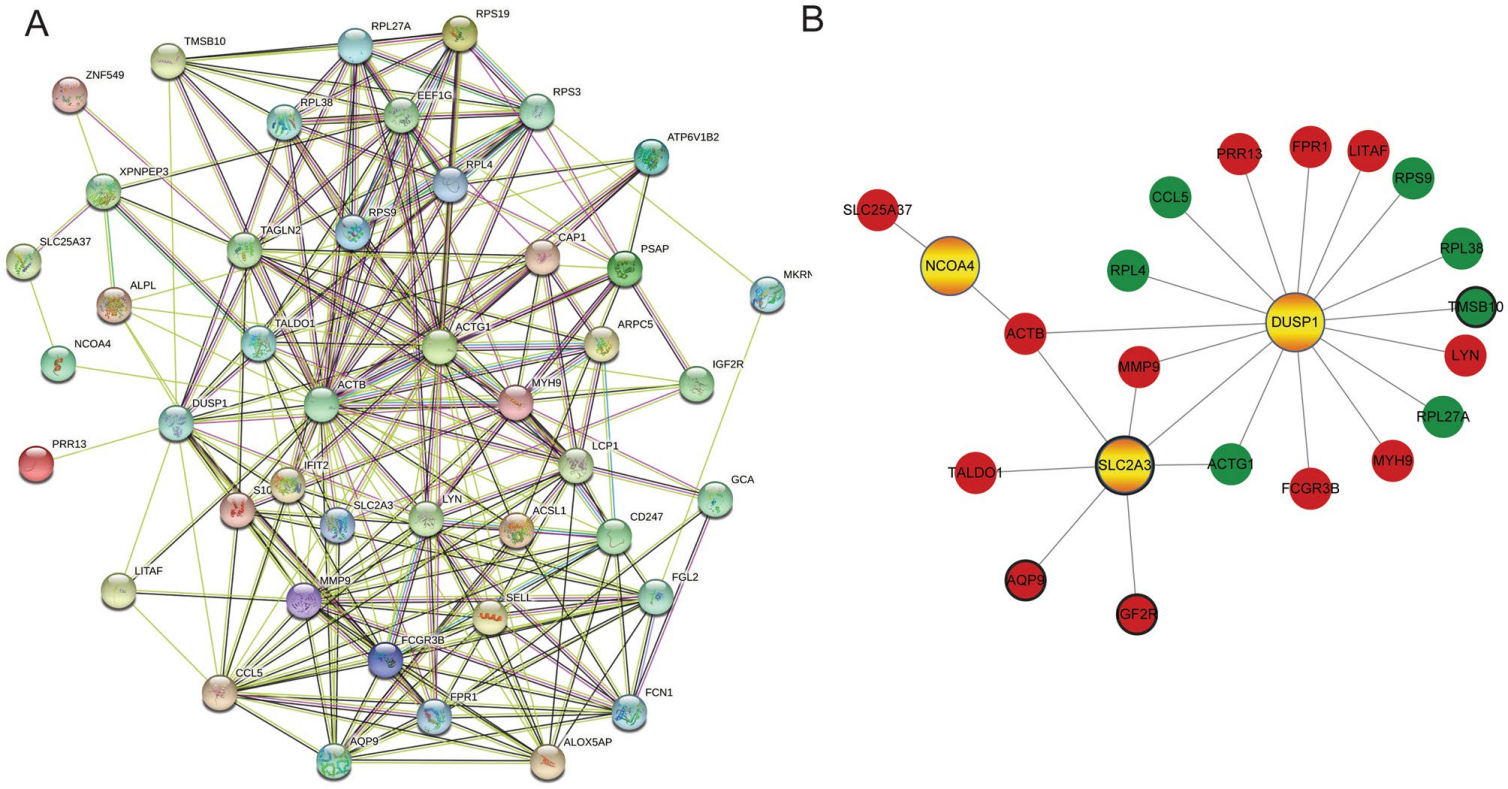
PPI analysis of mRNAs in the circRNA-miRNA-mRNA network in IS. (**A**) PPI network of 44 mRNAs; (**B**) PPI network of 3 ferroptosis mRNAs (colored by yellow). Red and green indicate up-regulation and down-regulation, respectively. Black border represents top10 up-regulated and down-regulated mRNA, respectively.

In order to explore the function of 44 mRNAs in the ceRNA network, GO and KEGG analysis was performed. In GO analysis, cytoplasmic translation (Fig. 4A), extracellular exosome (Fig. 4B) and structural constituent of ribosome (Fig. 4C) were the most significantly enriched biological process, cytological component and molecular function, respectively. In the KEGG analysis, some signaling pathways were identified, such as fluid shear stress and atherosclerosis (involved DUSP1) (Fig. 5). Detailed signaling pathways and involved mRNAs are shown in Table 3. In addition, the interaction between proteins encoded by mRNAs was explored (Fig. 6A). It is noted that 3 ferroptosis mRNAs of DUSP1, NCOA4 and SLC2A3 interacted with up-regulated actin beta (ACTB) (Fig. 6B).

**Table 3:** KEGG analysis of mRNAs in the circRNA-miRNA-mRNA network in IS.

Term	Count	*P* value	MRNAs
hsa03010: Ribosome	6	2.09E−04	RPL4, RPS9, RPS19, RPL27A, RPS3, RPL38
hsa05171: Coronavirus disease—COVID-19	6	0.001212	RPL4, RPS9, RPS19, RPL27A, RPS3, RPL38
hsa04613: Neutrophil extracellular trap formation	5	0.004295	FCGR3B, AQP9, FPR1, ACTB, ACTG1
hsa05130: Pathogenic Escherichia coli infection	5	0.004885	RPS3, MYH9, ARPC5, ACTB, ACTG1
hsa05418: Fluid shear stress and atherosclerosis	4	0.012803	DUSP1, MMP9, ACTB, ACTG1
hsa05110: Vibrio cholerae infection	3	0.013456	ATP6V1B2, ACTB, ACTG1
hsa04145: Phagosome	4	0.016265	FCGR3B, ATP6V1B2, ACTB, ACTG1
hsa04530: Tight junction	4	0.021516	MYH9, ARPC5, ACTB, ACTG1
hsa05120: Epithelial cell signaling in Helicobacter pylori infection	3	0.025394	LYN, CCL5, ATP6V1B2
hsa05100: Bacterial invasion of epithelial cells	3	0.030301	ARPC5, ACTB, ACTG1
hsa04810: Regulation of actin cytoskeleton	4	0.041305	MYH9, ARPC5, ACTB, ACTG1
hsa04666: Fc gamma R-mediated phagocytosis	3	0.046165	LYN, FCGR3B, ARPC5

### Expression validations of circRNAs, miRNAs and mRNAs in IS

**Figure 7:**
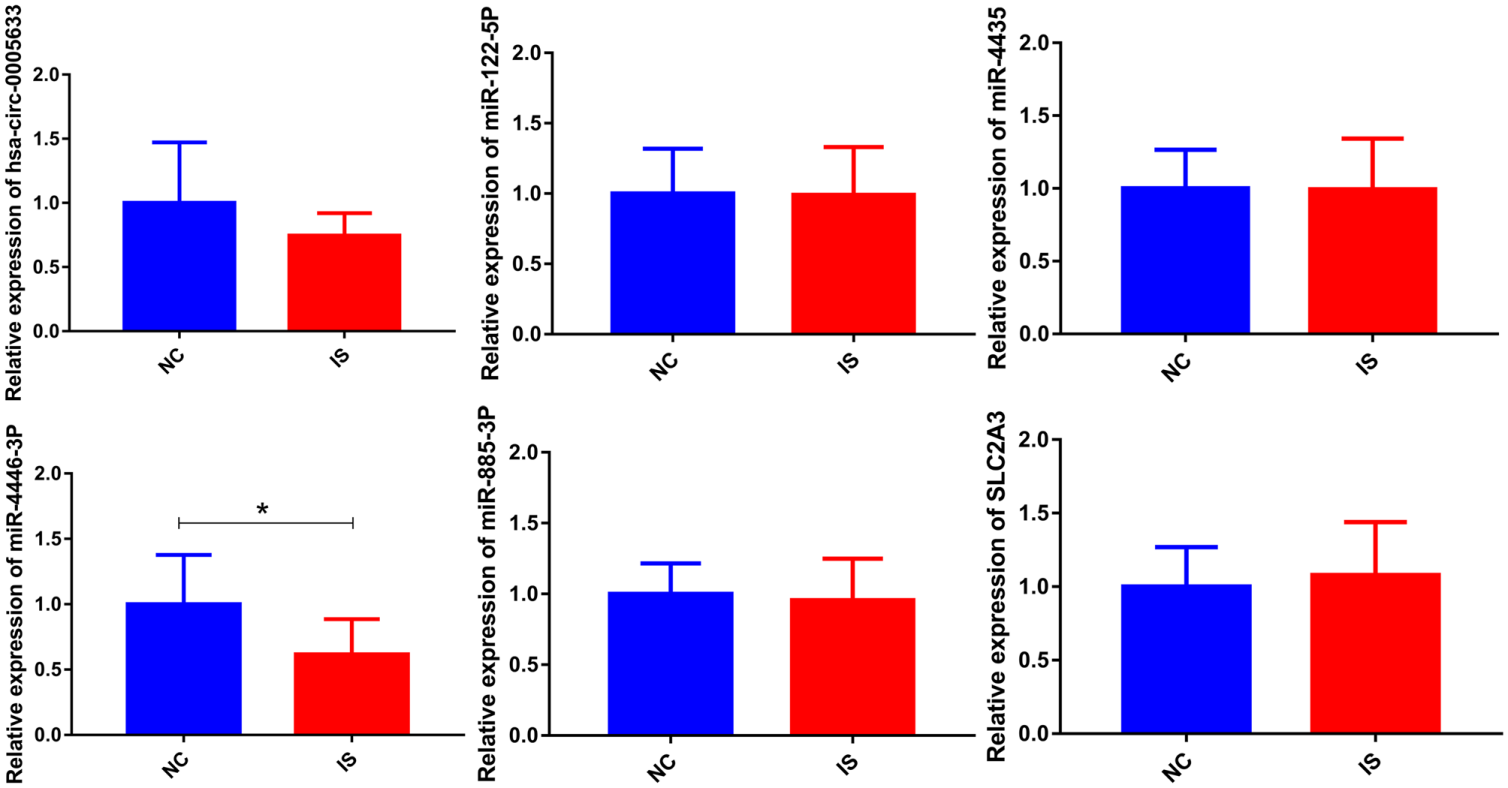
Expression validation of circRNAs, miRNAs and ferroptosis related mRNAs in IS. NC: normal controls; IS: ischemic stroke. *represents *p* value < 0.05.

**Figure 8:**
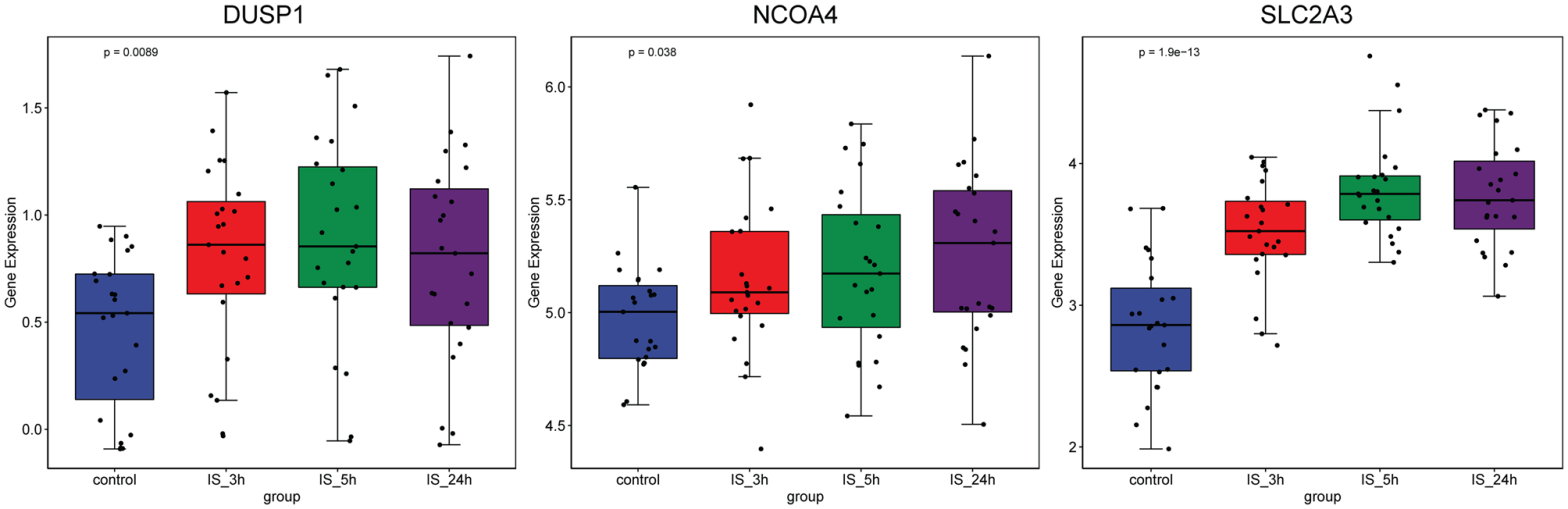
The expression of DUSP1, NCOA4 and SLC2A3 in IS after 3, 5 and 24 h of the attack. IS: ischemic stroke.

Firstly, 1 circRNA (hsa-circ-0005633), 4 miRNAs (hsa-miR-122-5p, hsa-miR-4435, hsa-miR-4446-3p, and hsa-miR-885-3p) and 1 ferroptosis related mRNA (SLC2A3) was used for expression validation by RT-PCR (Fig. 7). Hsa-circ-0005633, hsa-miR-122-5p, hsa-miR-4435, hsa-miR-4446-3p, and hsa-miR-885-3p were down-regulated, SLC2A3 was up-regulated in the blood samples of IS patients. The expression trends of hsa-miR-122-5p, hsa-miR-4435, hsa-miR-4446-3p, hsa-miR-885-3p and SLC2A3 were consisted with the informatics analysis results. In the GSE58294 dataset, the expression of DUSP1, NCOA4 and SLC2A3 was explored in blood samples of patients with IS after 3, 5 and 24 h of the attack (Fig. 8). DUSP1, NCOA4 and SLC2A3 were significantly up-regulated in IS after 3, 5 and 24 h of attack compared with normal controls. While, there was no significant difference in the expression of DUSP1, NCOA4 and SLC2A3 between 3 time points. It is indicated that DUSP1, NCOA4 and SLC2A3 play an important in the occurrence of IS.

## Discussion

Ferroptosis is an iron-dependent oxidative stress-induced cell death that has been shown to play an important role in IS^19–21^. Exploring the molecular mechanism related to ferroptosis in IS is helpful for disease diagnosis, prognosis and management. One study shows that Acyl-CoA synthetase long-chain family member 4 (ACSL4) exacerbates IS by promoting ferroptosis-induced brain injury and neuroinflammation^22^. Moreover, Circ-Carm1 can promote iron subsidence in acute cerebral infarction through the hsa-miR-3098-3p/ACSL4 axis^23,24^. Knockdown of Activating transcription factor 3 (ATF3) suppresses the progression of IS through inhibiting ferroptosis^25^. MiRNA-27a may aggravate brain tissue ferroptosis during IS by inhibiting nuclear factor erythroid-2-related factor 2 (Nrf2)^26^. The above studies suggest that exploring the molecular mechanism related to ferroptosis is helpful for the management of IS. At present, the molecular mechanism of ferroptosis related genes in IS is still unclear. Therefore, continuous identification of novel markers and potential mechanisms will help to diagnosis and management of IS. In this study, we found 3 up-regulated ferroptosis related mRNAs (DUSP1, NCOA4 and SLC2A3) in IS. Moreover, a circRNA-miRNA-ferroptosis related mRNA network was constructed based on DUSP1, NCOA4 and SLC2A3.

Hsa_circ_0071036 is present in the cytoplasm and play roles in post-transcriptional regulation^27^. In pancreatic cancer, hsa_circ_0071036 is significantly associated with unfavorable characteristics and prognosis, and high expression of hsa_circ_0071036 promotes tumourigenesis^28^. Hsa-miR-122-5p plays a key role modulating the cell proliferation, cell cycle and apoptosis^29^. In rats with transient cerebral ischemia, hsa-miR-122-5p is significantly down-regulated in cerebrospinal fluid and plasma^30^. In patients with recurrent ischemic, hsa-miR-122-5p is related to angiogenesis-related biological functions^31^. DUSP1 is involved in the synthesis and metabolism of fatty acids in the brain^32^. In atherosclerosis, DUSP1 plays a promoter and suppressor role^33,34^. It is found that DUSP1 is up-regulated in IS and may act as a new biomarker for the diagnosis and treatment of IS^35,36^. In this study, 4 novel circRNAs (hsa_circ_0071036, hsa_circ_0039365, hsa_circ_0079347 and hsa_circ_0008857) competitively bind to hsa-miR-122-5p to regulate the expression of DUSP1 in IS. It is indicated that these circRNAs, miRNAs and mRNAs may be involved in the process of IS.

Hsa_circ_0067717 is abnormally up-regulated in hepatitis B virus-associated hepatocellular carcinoma patients^37^. Hsa_circ_0001414 is down-regulated in patients with polycystic ovary syndrome^38^. Hsa-circ-0049637 is significantly correlated with neutrophils in acute IS^39^. Decreased expression of hsa-miR-4446-3p has been found in acute IS^40^. Hsa-miR-885-3p is involved in blood and nervous system development^41^. Hsa-miR-885-3p is down-regulated in acute IS patients with a worse prognosis^40^. SLC2A3, plays a crucial role in brain glucose uptake, is associated with the traumatic brain injury^42–44^. The increased expression of SLC2A3 has been observed in stroke-associated and asymptomatic carotid plaques^45^. Herein, some regulatory relationship pairs of hsa_circ_0067717/hsa_circ_0003956/hsa_circ_0013729-hsa-miR-4446-3p-SLC2A3 hsa_circ_0059347/hsa_circ_0001414/hsa_circ_0049637-hsa-miR-885-3p-SLC2A3 were identified in IS. It is suggested that the interaction between these circRNAs, miRNAs and mRNAs is involved in the development of IS.

Now, there is no related literature report on hsa_circ_0005633 and hsa_circ_0004479. Expression dysregulation of hsa-miR-4435 is associated with schizophrenia^46^. NCOA4-mediated ferritinophagy causes the degradation of ferritin and promotes the release of free iron, which is a key factor to cause ferroptosis. The deletion of NCOA4 significantly eliminated ferritin-mediated phagocytosis^47^. In the present study, hsa_circ_0005633, hsa_circ_0004479 and NCOA4 were up-regulated, hsa-miR-4435 was down-regulated in IS. The interaction between them may play roles in the process of cerebral ischemiareperfusion.

In the PPI analysis, DUSP1, NCOA4 and SLC2A3 interacted with ACTB. Lower methylation level of the ACTB is found in stroke patients^48^. Maybe, protein interactions between DUSP1, NCOA4, SLC2A3, and ACTB are involved in the molecular mechanism of IS. In addition, DUSP1 was involved in fluid shear stress and atherosclerosis. The maintenance of physiological laminar shear stress is important for normal vascular function^49^. Cipolla MJ et al. found that vascular shear stress affected cerebral auto-regulation and lead to stroke^50^. Atherosclerosis can cause stroke onset or recurrence, and blood flow-induced shear stress has become a key characteristic of atherosclerosis. It is speculated that DUSP1 may be involved in the process of atherosclerosis in IS.

Different researchers have not yet reached a general consensus on the time intervals for describing different stages of stroke^51–53^. MRI is an important method for diagnosing and evaluating stroke, but it exist serious challenges due to spatial and temporal resolution limitations^54^. The pathophysiology mechanism of blood–brain barrier permeability in different stages of IS is different^55^. Moreover, the immune response mechanism and molecular regulation mechanism of stroke in different stages are also different^56,57^. Therefore, exploring the expression of key molecules at different stages IS helpful to understand the molecular mechanism of the progression of IS. In this study, we analyzed the expression of DUSP1, NCOA4 and SLC2A3 at 3, 5 and 24 h after stroke based on the GSE58294 dataset. The results showed that DUSP1, NCOA4 and SLC2A3 were significantly up-regulated in IS after 3, 5 and 24 h of the attack. However, the expression differences between different time points of 3, 5 and 24 h do not seem significant. Therefore, it is speculated that DUSP1, NCOA4 and SLC2A3 may only be involved in the occurrence of IS.

In conclusion, 3 up-regulated ferroptosis related mRNAs (DUSP1, NCOA4 and SLC2A3) were identified in IS. Based on 3 ferroptosis related mRNAs, a total of 4 circRNA-miRNA-ferroptosis related mRNA regulatory relationship pairs were identified in IS, which may provide a new field in understanding the molecular regulatory mechanism in IS. Our study may provide a novel diagnosis and treatment option for IS patients at the molecular level. However, there are limitations to this study. Firstly, the sample size of the RT-PCR is small. Larger numbers of blood samples are further needed for expression validation. Secondly, the potential mechanism involved in the regulatory ceRNA network in IS is further needed to be investigated in animal model or cell experiments.

## Data Availability

The dataset supporting the conclusions of this article is included within the article. The database analysed during the current study are available in the GEO database, persistent accessible web link to database is https://www.ncbi.nlm.nih.gov/geo/. Accession numbers of the datasets used in the current study are GSE133768, GSE199942, GSE16561 and GSE58294. All data generated or analysed during this study are included in this published article.
